# 

**Published:** 1891-12-05

**Authors:** 


**BOVRIL** two years has established itself throughout Europe and America as the most perfect antidote, the most certain preventive and approved cure of the epidemic in all its stages. **BOVRIL** (the product of prime ox beef) furnishes to the system that stamina and vitality which can best counteract the influence of the scourge, and carries the feeble invalid through the climax of the attack to a rapid recovery.

Thursday I had a smart touch of Influenza, and have achieved such a rapid cure that it may be of service to your readers to know the means used. On Thursday I was totally incapacitated, on Friday convalescent, on Saturday quite well. Treatment each day—four dessert spoonfuls Cod-Liver Oil, one after each meal; four Teacups full of **BOVRIL** mid-way between meals, and last thing at night. I believe this will cure any one.—Yours, &c., (Signed) GEORGE S. HAZLEHURST, The Grange, Rock Ferry. J.P., Flintshire.

### THE HOSPITAL

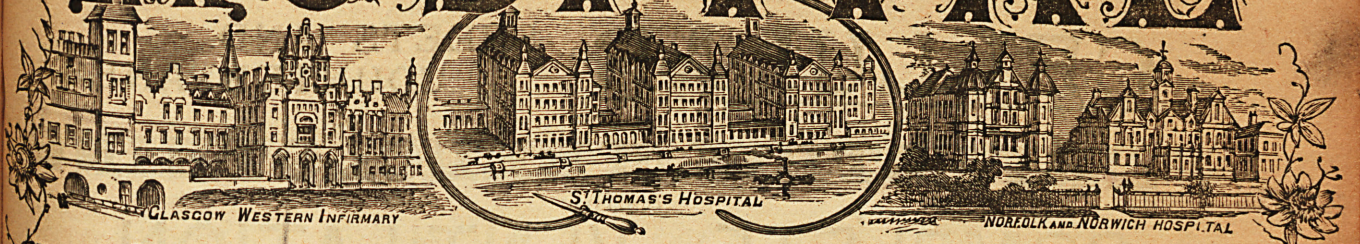

WITH EXTRA NURSING SUPPLEMENT.

No. 271. Vol. XI.] SATURDAY, DECEMBER 5th, 1891. [ONE PENNY.

